# Effective inter-organisational networks for Responsible Research and Innovation and global sustainability: a scoping review

**DOI:** 10.12688/openreseurope.13796.2

**Published:** 2022-07-20

**Authors:** Danielle Martine Farrugia, Silvia Leonor Vilches, Alexander Gerber

**Affiliations:** 1 University of Malta, Msida, MSD 2080, Malta; 2 International Consortium of Research Staff Associations (ICoRSA), Cork City, Ireland; 3 Department of Human Development and Family Studies, Auburn University, Auburn, United States, Auburn, USA; 4 Science communication, Rhine-Waal University, Kleve, Germany

**Keywords:** RRI, SDGs, network theory, inter-organisational networks, governance, trust

## Abstract

**Background: **Achieving the United Nations Sustainable Development Goals (SDGs) is beyond the capacity of any single organisation. The model for Responsible Research and Innovation (RRI) includes principles of engaging stakeholders and suggests that an engaged, multi-sectoral approach hold promise to mobilise humanity to solve complex and urgent global issues.

**Methods: **This scoping review explores the characteristics of effective and sustainable inter-organisational networks for fostering RRI in service of the SDGs. An inductive-deductive search of prior studies (1990-2020), with the exception of Benson’s (1975) seminal work was conducted, which focused on strategies to initiate and maintain inter-organisational networks relevant to the implementation of RRI and/or SDGs. The search began with themes derived from prior network theory, focusing on: (a) the type and function of networks; (b) the aims and vision; and (c) the relationships between networks and network members. In total, 55 articles on inter-organisational network theory were included for the final analysis.

**Results: **Results are reported under themes of: (1) Effectiveness, Sustainability, and Success; (2) Governance and Management; and (3) Network Relationship. Network structures, forms of management and funding are linked to sustainable networks. Potential threats include power imbalances within networks, and internal and external factors that may affect relationships at network and community levels. Few studies examine diversity or cultural viewpoints. Studies highlight the benefits of networks such as enhancing knowledge sharing among researchers, practitioners, and other stakeholders.

**Conclusions: **The effectiveness of the managerial structure may be observed as outputs of the intention and values of an inter-organisational network. Our review demonstrates that a global inter-organisational network approach is achievable. Such a network would have many benefits, including allowing organisations to be responsive and flexible towards change and innovation.

Keywords

RRI, SDGs, network theory, inter-organisational networks, governance, trust

## Introduction

Although research provides evidence-based insights and theory, the European Commission (EC) states that scientific endeavours need to be integrated with the social, economic, cultural, and political spheres to enhance creativity in research and innovation processes, ensuring that outcomes will be more socially relevant ( EC, 2020a, Jul. 21). This approach has been captured in the development of the Responsible Research and Innovation (RRI) framework. Furthermore, RRI focuses on the so-called ‘pillars’ or five thematic elements: public engagement, open access, gender, ethics, and science education ( EC, 2020b, Dec 7). While RRI may be applied to any domain of activity, the Sustainable Development Goals (SDGs), adopted as part of the 2030 Agenda for Sustainable Development (2030 Agenda) by the 193-Member United Nations General Assembly in 2015, set the stage for a global challenge to mobilise in achieving the SDGs ( UNESCO, 2015).

In Europe, there are a number of partly funded networks with an RRI dimension focusing on and/or implementing RRI pillars or process dimensions to innovate responsibly. Such networks tend to operate at various levels: regional, national, European or on a global scale ( Dalton *et al*., 2020, p. 11). While previous literature has struggled with identifying generalisable and clear predictors for a network to be successful ( Kiefhaber, 2016) our review does highlight a number of very relevant opportunities and risks.

### Network theory

Networks are defined as interchanges among the people who form them, providing a space for social interaction ( Valencia & Cázares, 2016, p. 2). These interactions allow for a group of people to contribute or work together towards a common goal or to find a solution to a problem ( Valencia & Cázares, 2016, p. 2). Although a group of actors/stakeholders can be considered a network, the term also refers to how the actors/stakeholders operationalise and conceptualise the network ( Isett *et al*., 2011, p. 160). A network may also represent an important form of multi-organisational governance ( Provan & Kenis, 2008, p. 229). Such interactions may be facilitated by communication technologies ( Lehoux *et al*., 2018, p. 10).

Many definitions of networks in general and inter-organisational networks exist ( Popp *et al*., 2014, p.10). Networks share some common elements including social interaction, relationships, collaboration, action, trust, and cooperation ( Provan *et al*., 2007, p. 481). Bryson *et al.* (2006, p. 44) suggest that an effective network aims towards cross-sectoral collaboration and partnership involving different entities and communities. Networks can be organised in informal or formal hierarchical systems ( Provan *et al*., 2007, p. 504; Provan & Kenis, 2008, p. 234).

No single theoretical approach explains the multitude of different approaches to studying networks, however, Provan *et al*. (2007, p. 482–483) suggest two different, albeit complementary, approaches to studying the effect of networks. The first approach starts from the level of the individual actor/stakeholder (micro-level), while the second focuses on the network level (macro-level). This first category (micro-level) explores the relationships and impact of individual organisations on each other as well as how a network as a whole affects the individual organisations. The second, macro-level, approach is used to explore how the actions of individual organisations might affect network-level outcomes and whole network or network level interactions such as structures, stability, and effectiveness. In addition, as noted by Provan & Kenis (2008, p. 229), network level outcomes are also affected by network conditions, referred to as network functioning.

Actor-Network Theory (ANT) offers a second approach to studying networks. ANT is a theoretical and methodological approach to social theory involving both human and non-human actors, also referred to as actants, who are connected in a network of meaning ( Sayes, 2014, p. 134–135). ANT provides a useful starting point to understand the complexity of interactions between human and non-human actants in organisational networks. Applications of ANT in planning contexts suggest that ANT provides a useful lens for exploring the flows of power, and boundaries between human and non-human actants ( Rydin & Tate, 2016, p. 14–15). ANT offers the opportunity to analytically understand how some of the interactions of human actants affect non-human actants and vice-versa, although this is still problematic ( Sayes, 2014, p. 140). Klijn & Koppenjan (2012, p. 2, p. 11–12) note strong development in the area of network governance, which indicates an accepted area of practice and research ready for development of theoretical constructs. Montenegro & Bulgacov (2014, p. 111,114) assert that network governance theory may be made more robust with the inclusion of non-human actants due to the self-governing nature of networks, in which groups are defined, not by occupational role but by the process of forming and action. In RRI, in which the structure of domains such as public and private, entrepreneurial and government, and citizen and environment all interact, the capacity to act is constrained and defined by the boundaries of different perceptions and capabilities, suggesting that ANT provides a useful analytic lens. While generally useful, our scoping review does not specifically include literature about ANT.

Building a network for implementing RRI practices appears particularly challenging under the umbrella concept of openness of networks, given RRI’s various ways of implementation, and its distributed governance, policy and practices ( Dalton *et al*., 2020, p. 11). As a result, the determinants of what makes networks effective or successful are largely unclear. As described by Provan & Kenis (2008, p.230), network effectiveness can be described as attaining positive outcomes at the network level that are better achieved by participants from individual organisations working together.

### Applying network theory to RRI implementation

The conceptual framework of RRI was developed in European science policy contexts, to explicitly address global concerns. Over the past decade, RRI has changed ( Owen *et al*., 2021, p.11). In 2014, it was described as a set of values or a process where all societal actors work towards aligning research and innovation outcomes to the values, needs, and expectations of society ( EC, 2014, p. 2). Another definition emphasises the difficulty of the ethical implications of involving different groups within society at every aspect of the research process ( von Schomberg, 2013, p. 19). Currently, the EC defines it as “...an approach that anticipates and assesses potential implications and societal expectations concerning research and innovation, intending to foster the design of inclusive and sustainable research and innovation” ( EC, 2020b, Dec 7). RRI can therefore be seen as an approach for creating institutional change ( Stilgoe *et al*., 2013, p. 1576). Global contexts change depending on the needs of society, including RRI as applied in the South and island nations under climate threats.

There are four process dimensions of innovating responsibly: anticipation, reflexivity, inclusion and responsiveness ( Stilgoe *et al*., 2013, p. 1570). The anticipation dimension involves different stakeholders, (e.g. researchers, policymakers, and others) in foreseeing issues in the development of science and technology ( Macnaghten, 2020, p. 13; Stilgoe *et al*., 2013, p. 1570). The reflexivity dimension includes not only the evaluation of scientists’ and/or researchers’ own responsibility towards innovation, but also the need for institutional reflexivity. The framework implies that institutions have the responsibility to do more than just reflecting on their value systems, but also to develop a reflexive capacity for practicing science along with practicing innovation ( Macnaghten, 2020, p. 1571; Stilgoe *et al*., 2013). Focusing on the dimension of inclusion along with innovation in science also means moving away from an approach of one-way science communication to considering how to incorporate different public sectors ( Stilgoe *et al*., 2013, p. 1575). Finally, utilising the RRI framework also requires the capacity to be responsive (fourth dimension) when engaging with citizens and other stakeholders ( Macnaghten, 2020, p. 16; Stilgoe *et al*., 2013, p. 1572). As Ludwig & Macnaghten (2020, p.29,37) point out, an RRI approach cannot ignore or be dissociated from cultural norms and values, as RRI is interpreted differently depending on a country’s specific societal needs and values. These interactions can be conceptualised through a political economy of science governance for inclusion of diverse stakeholders’ viewpoints on the purpose of intended innovation ( Macnaghten, 2020, p. 15).

Scholars also identify the need to broaden the scope of RRI and expand beyond a focus on research to include how knowledge and ideas are implemented ( Jakobsen *et al*., 2019, p. 2333; Owen *et al*., 2021, p. 10). One challenge is that actuating the RRI framework and implementing SDGs requires resources, including funding. The last three European framework programmes for funding research and innovation have instituted changes in processes so that RRI principles are required, together with implementing RRI practices into organisations, and drafting of national level policies ( Gerber *et al*., 2020, p. 709). Numerous large-scale EU-funded RRI projects, (including the Responsible Research and Innovation Networked Globally [ RRING] project), signed a joint declaration, urging the EC to make RRI a key objective of the then upcoming Horizon Europe framework programme. The task was to mainstream the RRI approach throughout the programme and to provide specific resources to strengthen the RRI knowledge base ( Gerber *et al*., 2020, p. 710).

Another challenge is the multiplicity of engaged sectors. The EC identifies engagement of societal actors as important to the uptake of scientific work and public science, the process of implementation consistent with values, and participating in establishing priority outcomes ( EC, 2020b, Dec 7). However, a difficulty lies in how to involve societal actors in decisions revolving around evidence-based policy-making. Citizen science organisations (CSOs) and research empowered by citizens, scientists and researchers, together with technology, provide a potential platform for stakeholders to engage with science ( Göbel *et al*., 2016, p. 9). Such platforms are used to democratise scientific inquiry and tackle societal challenges ( Hajibayova, 2020, p. 924; Levikov *et al.*, 2020, p. 22). Citizen science research, conducted through non-governmental organisations (NGOs) or other European projects and organisations, is seen to have great potential in engaging various publics especially in the context of RRI and the SDGs, in building expertise, and representing community interests ( Göbel *et al*., 2016, p. 14). The inclusion of citizen science raises questions about diverse sources of innovation. Inter-organisational networks can assist in creating an organisation that is flexible towards change, innovation, and responsiveness ( Popp *et al*., 2014, p. 70). However, since stakeholders have different motives and resources, RRI principles, as applied into innovation processes beyond those that are research-based, remain unclear ( Jakobsen *et al*., 2019, p. 2332).

### Applying network theory to an RRI/SDG context

Each of the 17 SDGs engages a multiplicity of domains of action ( UNESCO, 2015). Using good health and well-being (SDG3) as an example, the concept of RRI can be applied to support solutions through industry and innovation (SDG9), as well as a holistic approach to health through health literacy and quality education (SDG4), and/or having clean water and sanitation (SDG6) ( Lehoux *et al*., 2018, p. 10). Other related SDGs encompassing health are those relating to environmental sustainability such as affordable clean energy (SDG7) and climate action (SDG13). Implementing the multiple objectives in even one SDG implies a decentralised model of governance. Increasing the difficulty of applying SDGs on a universal scale is the absence of tools and frameworks to assist with strategic and transformational change ( Grainger-Brown & Malekpour, 2019, p. 15). The challenge invoked by considering multiple domains of action elevates the complexity of moving forward to addressing goals, and highlights the opportunity potentially offered by inter-organisational networks.

There are publicly funded networks that adopt an RRI framework or aim to achieve the SDGs. For example, the Organisation for Economic Co-operation and Development (OECD) is an international network that works to build better policies, and is funded by its European and other member countries. Networking generally happens on a regional, national, global, or European levels through conferences, workshops, and events ( Rai, 2003). Different inter-organisational networks, including social, research, and trade networks, promote a particular position or agenda. For example, certain networks promote jobs ( Chua & Wellman, 2015, p. 906), others enable conversations concerning gender and governance across national borders ( Rai, 2003, p. 59), and a United Nations inter-agency network promotes the realisation of gender equality ( Inter-Agency Network on Women and Gender Equality, 2004, p. 3).

Despite the potential offered by networks, the effectiveness of institutional networks for sustaining the implementation of RRI principles has not yet been explored using network theory. As mentioned previously, having an effective network means that there is a coordinated effort between participants from individual organisations ( Provan & Kenis, 2008, p. 230). Network effectiveness can also be viewed from the lens of structural characteristics and contextual factors, including network integration, external control, system stability, and environmental resource munificence ( Provan & Milward, 1995, p. 1). This paper investigates the theoretical underpinnings in network effectiveness, specifically the themes of governance and management of inter-organisational networks, as well as relationships that ensue at both network and community level, and whether or how they may apply to RRI/SDG based networks. We explore the following questions:

- How is effectiveness understood and discussed in inter-organisational network theory?
- Which factors from inter-organisational network theory help us understand how to manage, govern and maintain an effective, sustainable, and successful RRI/SDG network?

## Methods

A scoping review approach ( *cf* Tricco *et al*., 2018) was used to search for articles on networks that explore the values and practices of RRI/SDG discourses and policies. Scoping reviews are useful when a systematic search is desired, but the existing literature is heterogeneous, new or evolving, or does not use consistent criteria, preventing comparison of findings between studies ( Arksey & O’Malley, 2005, p. 20). A scoping review is used to identify gaps in research and inform the planning of future research ( Tricco *et al*., 2018, p. 467). A protocol per Tricco *et al*. (2018) has been provided, as requested by the editors, but this was developed as an exploratory scoping review. Scoping reviews are also used to establish terminology, or what is known as terminology warrant, thereby helping to advance a field of study by identifying discrepancies or congruence in the way concepts are described and approached ( Grant & Booth, 2009, p. 93; Webster & Watson, 2002, p. i).

Initially, an expert group within the working group for task 7.1 in the Horizon 2020 project RRING suggested literature based on the project’s underlying aims, one of which is to establish a potential RRING community ( Dalton *et al*., 2020, p.11–13). The expert working group reviewed a selection of 28 articles to develop search criteria ( Dalton *et al*., 2020, p.12). The criteria include: (a) the type and function of networks; (b) the aims and vision; and (c) the relationships between networks and network members. These three criteria reflect applied interests relating to RRI/SDG including networks that: (a) are global, intercultural, and have diverse members; (b) engage in knowledge exchange and collaboration (aims and vision); and (c) develop trust and collaborations required for the sustainability of the network both at network and community level ( Dalton *et al*., 2020, p.11–13).

A set of search strings (see Table 1) were developed that reflect the three characteristics. Initially, these search strings were chosen because our preliminary review presented different variants of the same term and the variants broadened the search. To ensure a comprehensive coverage of the field of network theory, synonyms and alternative phrasings were included as Boolean logic gates to the search strings to yield a richer set of sources: effectiveness, outcomes, performance, consequences, impact and results. A longer date range (1990 and 2020) was chosen to include works of early scholars in this field, (e.g. Provan & Kenis, 2008; Provan & Milward, 1995), as well as Benson (1975)’s work, which lies outside of this date range.

**Table 1.** Search strings utilised for inclusion of articles published between 1999 and 2020.

**Search Strings**	organisational networks
	organizational networks
	Inter-organisational coalitions
	Inter-organisational networks
	public private partnerships
	community partnerships
	organisational partnerships
	intergovernmental
	interlocal
	interagency
	collaborative initiatives
	alliances
	consortia
	multi-organisational
**And/OR Boolean gates** **applied to search strings**	effectiveness
	outcomes
	performance
	consequences
	impact
	results

Two databases were used to search for articles. Web of Science is an academic publication database, which draws from multiple disciplines and screens for peer-reviewed publications. The search domain chosen from within Web of Science was social sciences, focusing on public administration and management as subject areas. In addition, social science databases were searched more broadly using the ProQuest Social Science Database and Sage knowledge (ProQuest) available through the University of Malta (UM) library. The search strings were applied to the databases between 16th July and 23rd July 2020, yielding 221 results: 209 from Web of Science and 12 from the UM Library. Other additional material such as reports (n=15) were gathered from Google between the months of July 2020 to December 2020 using the search strings (See Table 1).

**Figure 1.**
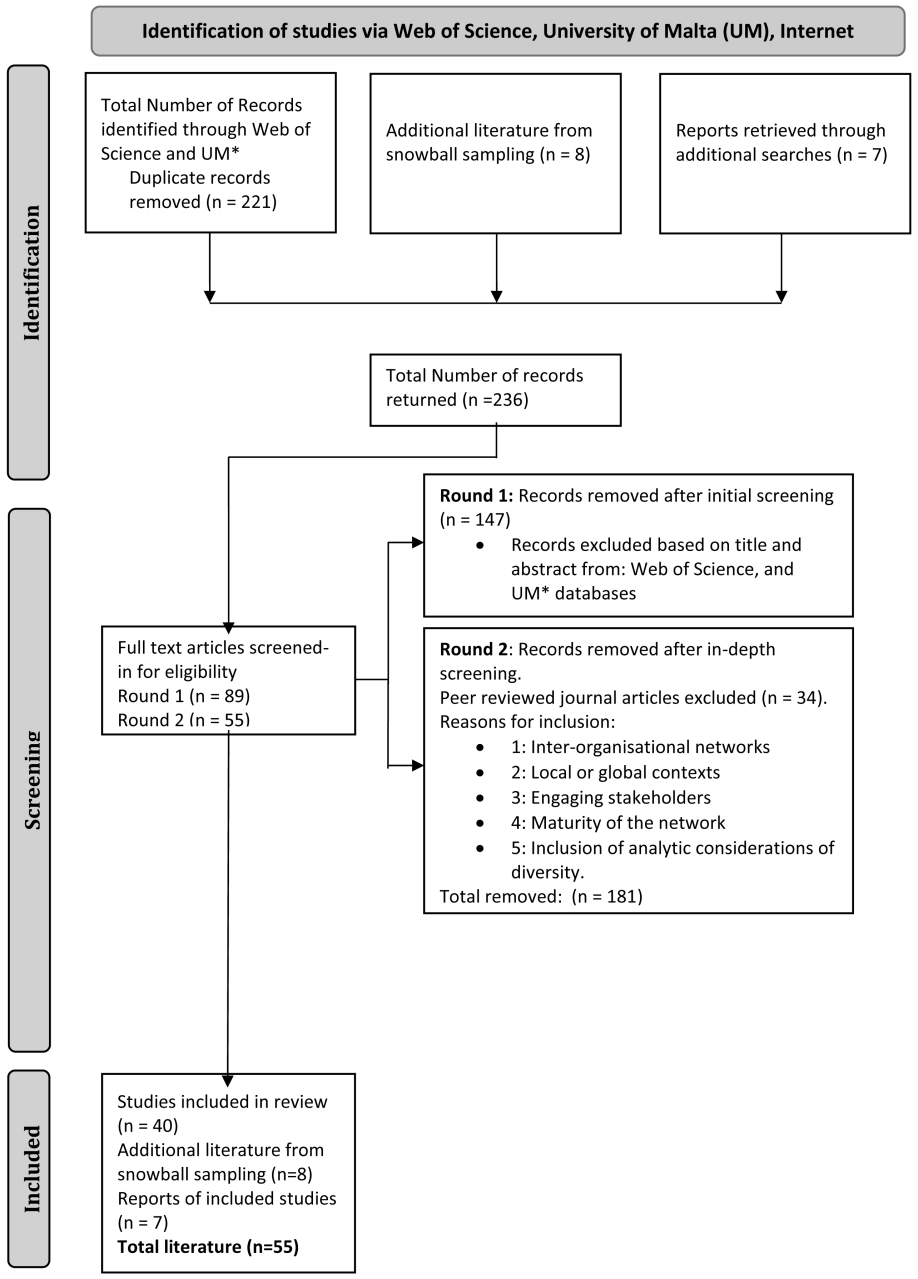
PRISMA-ScR Flow Chart for RRI Network Studies.

In forming the inclusion and exclusion criteria and to counteract the limitation that there is no specific literature on networks focusing on RRI or SDG, the ‘pillars’ of RRI and SDG principles were used to organise the search. We developed the inclusion and exclusion criteria from three of the eight characteristics proposed by Popp *et al.* (2014). The eight characteristics are: (1) key concepts and characteristics; (2) network types and functions; (3) network governance models, leadership and management, and structure; network membership models; (4) network funding models; (5) budget models and roadmap models; (6) risk management models; (7) network implementation; and (8) evolution models. To minimise the risk of excluding relevant literature, two reviewers (Danielle Martine Farrugia and Louisa Grabner) conducted our filtering approach in two parts (cf. Waffenschmidt *et al*., 2019). In part one we filtered articles from the Web of Science and UM databases based on their titles and abstracts (74 articles). Part two was more in depth (40 articles), where the entire paper was scanned for the criteria explained above, in order to include details on whether the articles focused on inter-organisational networks, engaging stakeholders, local or global contexts, the maturity of the network, and inclusion of analytic considerations of diversity. After the second screening, 40 articles were included together with seven reports. Eight additional articles from snowball sampling, recommended either through the literature explored or from the expert panel were added for a final list of 55 articles (See Figure 1, Farrugia *et al*., 2021a).

### Analytic approach

The scoping review drew a heterogeneous set of publications, so a qualitative thematic analysis process was used ( Boyatzis, 1998, p. 1–28) to identify patterns in the information and organise interpretation. We used a combined deductive and inductive process. First, in an initial deductive step, we identified patterns related to effectiveness, sustainability and success of networks. The results were categorised under three main themes: (1) effectiveness, sustainability, and success; (2) governance and management; and (3) network relationships (see Table 2). Following the initial deductive approach, an inductive process was used to further develop and generate the sub-codes and codes. A codebook was developed and used to systematise further coding (see Table 3) using ATLAS.ti analytic software. Other alternative open-source software packages such as Taguette software can be used. The analysis resulted in the development of several categories within each thematic area of investigation (see Table 3).

**Table 2.** Main Themes and Categories.

Theme 1: effectiveness, sustainability, and success	Theme 2: governance and management	Theme 3: network relationships
**Main categories**		
1.1 Network effectiveness	2.1 Type of network	3.1 Memberships
1.2 Network success	2.2 Network governance	3.2 Stakeholders & Actors
1.3 Network sustainability (integrated with 1.1 and 1.2)	2.3 Management	3.3 Relationships
	2.4 Funding	

**Table 3.** Hierarchy of themes and their main categories, codes and sub-codes.

**1. Theme 1: Effectiveness, Sustainability,** ** Success** **1.1 Network effectiveness** 1.1.1 External control 1.1.2 Non-fragmented external control 1.1.3 System stability 1.1.4 Performance 1.1.5 Aims/goals/vision 1.1.6 Other **1.2 Network sustainability** 1.2.1 Barriers 1.2.1.1 Cultural 1.2.2 Best practice 1.2.3 Challenges 1.2.4 Evolution **1.3 Network success** 1.3.1 Age 1.3.2 Membership 1.3.3 Density 1.3.4 Evaluation 1.3.5 Services 1.3.5.1 Networking 1.3.5.2 Resources 1.3.5.3 Support 1.3.6 Diversity 1.3.7 Knowledge 1.3.8 Innovation
**2. Theme 2: Governance and Management** **2.1 Type of network** 2.1.1 Inter-organisational 2.1.2 Citizen Science organisations 2.1.3 Network creation 2.1.4 Network evolution **2.2 Network governance** 2.2.1 Administrative organisation 2.2.2 Brokering 2.2.3 Shared 2.2.4 Mandated networks **2.3 Management** 2.3.1 Hub model 2.3.2 Orchestration 2.3.3 Function 2.3.4 Roles 2.3.5 Leadership 2.3.6 Other **2.4 Funding** 2.4.1 Downsides 2.4.2 Subsidies 2.4.3 Donations and sponsorship 2.4.4 Membership fees 2.4.5 Research grants
**3. Theme 3: Network Relationships** **3.1 Memberships** 3.1.1 Members' interests 3.1.2 Membership 3.1.3 Inclusive **3.2 Stakeholders & Actors** 3.2.1 Diversity 3.2.2 Network size **3.3 Relationships** 3.3.1 Power 3.3.2 Conflicts 3.3.3 Trust 3.3.3.1 Resiliency 3.3.3.2 Specificity 3.3.3.3 Credibility 3.3.4 Communication 3.3.4.1 Social media 3.3.5 Collaboration 3.3.6 Reputation 3.3.7 Social capital

## Results

Three sources of evidence were applied to the search strings (See Table 1); Web of Science, a selection of social science databases from the UM library and internet available reports. Results (n=55) include peer-reviewed articles (n=40), additional literature from snowball sampling (n =8), and reports (n=7) (See Figure 1). The findings are reported under three overarching themes: (1) effectiveness, sustainability, and success in inter-organisational networks; (2) governance and management of networks, structure, and the concerns of stakeholders; and (3) network relationships between network organisations and membership issues. Multiple screened-in literature were used for each of the three themes and were not exclusive to one or the other.

### Theme 1: network effectiveness, sustainability, and success

Various scholars have looked at multiple aspects to understand the effectiveness of a network ( Raeymaeckers & Dierckx, 2012, p. 486), or whether a network can be considered to be successful and sustainable. The included literature demonstrated connections between the terms effectiveness, sustainability, and success, which are used frequently and sometimes interchangeably. As a result, category 1.2 (sustainability) was removed as a stand-alone category as it overlaps with both category 1.1 (effectiveness) and category 1.3 (success) and integrated in the effectiveness and success categories.

***Category 1.1: effectiveness.*** The specific role of the network seems to contribute to the effectiveness of network activities and the determining factors towards the success and sustainability of the network. The determinants of network effectiveness are difficult to identify due to the intricate and complex relationships that exist in and between networks and network studies. A lack of exchange between different disciplines in network studies results in difficulty in determining factors for network effectiveness ( Dalton *et al*., 2020, p. 11). As suggested, while it is difficult to generalise and have a set of predictors for a network to be considered successful ( Kiefhaber, 2016), our review points out some very relevant opportunities and risks in setting up the foundations of such a community. Such a community is able to promote, disseminate and mobilise mutual learning in RRI and SDGs ( Dalton *et al*., 2020, p. 37).

The effectiveness of networks lies in achieving positive outcomes through collaborations that cannot be achieved by acting independently ( Grueso Hinestroza, 2015, p. 3; Provan *et al*., 2007, p. 482; Raab *et al*., 2015, p. 484). This is also true for dealing with solving large public health problems ( Loitz *et al*., 2017, p. 2). As explained in the introduction, this is similar to solving health issues when applying certain SDGs such as having clean water and sanitation (SDG6) ( Lehoux *et al*., 2018, p. 10). Performance is linked to how effective inter-organisational networks are ( DeWever *et al*., 2005, p. 1526). The outcomes that define network effectiveness can be said to be related to the function being evaluated under very specific characteristics, which make that network unique ( Popp *et al*., 2014; Provan *et al*., 2007). In an effort to create an integrated framework of an effective network, Turrini *et al.* (2010, p. 534) draw out concepts and their variables to understand the positive or negative impacts of network performance on three levels: client, community and network.

Mandell & Keast (2008, p. 720–722), identify three levels of effectiveness when dealing with inter-organisational networks: environmental, organisational, and operational. The environmental level includes all relevant stakeholders together with their impact on the network. The organisational level refers to the impact of the structural characteristics of the variety of networks. The operational level refers to the interactions between different participants.

A network’s age seems to be an important indicator of a network’s effectiveness ( Raab *et al*., 2015, p. 484), as whether a network is effective over a long period indicates the network’s sustainability. Raab *et al.* (2015, p. 505) highlight that it may take at least 3 years for healthcare and social service networks to provide a good service to clients. On the other hand, a mature network cannot be considered a successful or sustainable network unless the services are evaluated to understand whether members of the network need them ( Raab *et al*., 2015, p.485). However, age is not the only determining factor indicating a network’s effectiveness, even if it is an important criterion ( Raab *et al*., 2015, p.503, 504).

While evaluating effectiveness at the network level is important, this may not satisfy funders if it is not linked to organisational and members’ outcomes ( Popp *et al*., 2014, p.75). Although funding is necessary, it is not sufficient to ensure a network’s sustainability.

Järvensivu & Möller (2009) claim that the four management functions create value for their members, and are required for the success of any network. These four management functions include the managers’ need to plan, organise, lead and control the planning of the organisation ( Järvensivu & Möller, 2009). However, there is no single set of criteria on how to manage a network.

Ascertaining the factors influencing organisational effectiveness of a network varies across multiple aspects. First, effectiveness may be dependent on how effective the leadership of a network is ( Mcguire & Silvia, 2009, p. 34), or alternatively, how well leadership communicates with the network. Effectiveness could also depend on the services the network provides to its members, in terms of added value to their career and modes of engagement with different stakeholders, which could not be achieved on an individual level ( Provan & Kenis, 2008, p. 231). The review demonstrates that, although effectiveness is important, there is little consensus on what constitutes organisational effectiveness or how to measure it. This is because networks can be measured at the organisational or network level ( Provan & Kenis, 2008, p. 229; Raab *et al*., 2015, p. 484).

***Category 1.2: success.*** Success in a network is highly dependent on the function of the network, and whether success is measured by the network’s effectiveness at the network level, or by the services it provides to its members. DeWever *et al*. (2005, p. 1525), who examine the functioning of inter-organisational networks, suggest that a successful network depends on the structural and relational dimensions of social capital. The OECDb (2001) describes social capital as the sharing of values, norms, and understandings assisting collaboration between and among groups.

DeWever *et al.* (2005) describe social capital as a multidimensional construct that can facilitate resources available within or through the network of relationships of an organisation. They suggest three factors, based on social capital and network theory that affect network success: network configuration, the level of configuration, and the interaction between trust and network configuration in acquiring resources for the survival of the network ( DeWever *et al*., 2005, p. 1525). For Levén *et al.* (2014), success factors of high value innovation networks were having diverse partners, financial leverage, third-party gatekeepers, and partners who were proactively engaged. However, since collaborations are subject to competitive and institutional pressures that may affect long-term sustainability, these pressures may affect the success of a network ( Bryson *et al*., 2006, p. 45).

Other factors that influence the effectiveness, sustainability, and success of a network are the ability to innovate ( Barnett *et al*., 2011, p. 1) and the level of trust in relationships at the network level, between partners, and network members ( Antivachis & Angelis, 2015, p. 584). Another determinant of success is provided through an evaluation of whether a network has reached its goals ( Provan *et al*., 2007, p. 397–398; Raeymaeckers & Dierckx, 2012, p. 493–494). The characteristics of network success rely on the structural and relational aspects of the network, as well as evaluating the network.

In summary, effectiveness, success and sustainability are closely interwoven indicators of network viability and achievement. All three categories can be used to evaluate the functioning of a network. Networks may assist in facilitating collaborations ( Grueso Hinestroza, 2015, p. 3; Provan & Kenis, 2008, p. 243; Raab *et al*., 2015, p. 479), which can be measured through positive or negative impacts of network performance. The age of the network's existence indicates whether a network can be considered sustainable, as discussed above. In addition, other factors that influence the effectiveness, sustainability, and success of a network are the ability to innovate ( Barnett *et al*., 2011, p. 1) and the level of trust in relationships at the network level, between partners, and network members ( Antivachis & Angelis, 2015, p. 584). The following sections will explore the other two main themes in this scoping review: Governance and Management, and Network Relationships at the network and community level.

### Theme 2: governance and management

The main categories within the theme of Governance and Management were: the type of network, network governance, management and funding. Further codes and sub-codes were created while coding and the results from the screened literature is explored below.

***Category 2.1: type of network.*** The terms ‘function’ and ‘types’ are used interchangeably in the literature on network theory ( Popp *et al*., 2014, p.30). Networks may have more than one function, such as information sharing or service delivery in for example, healthcare networks ( Provan & Kenis, 2008, p. 230) or in promoting participatory research activities in citizen science networks ( Göbel *et al*., 2016, p. 12). However, these functions may well be seen as outcomes ( Provan & Kenis, 2008, p. 229), services, and added value for the members. Functions of such services may be primary or multiple (Popp *et al*., 11). Therefore, the distinction between functions, understood as types, and outcomes of networks are useful when evaluating a network.

***Category 2.2: governance.*** The choice of governance in a network depends on the type/function of the network, which will influence the decisions on how the network is managed ( Provan & Kenis, 2008, p. 237). We noted that as with types of networks, governance and management structures are also intertwined, as governance structure affects the management of the network and in turn how effective, sustainable, and successful the network is or can be.

Three ideal modes of network governance are presented in Provan & Kenis (2008, p. 237) that can lead to effective outcomes are: the self-governed network, the lead organisation model, and the network administrative model. A self-governed network model is comprised of different participating organisations coordinating the activities together, while a lead organisation model is a network that is governed by one organisation, usually the one with the most interest in the network ( Raab *et al*., 2015, p. 482). The network administrative model has a separate organisation that is set up to coordinate and facilitate activities and represent the network externally ( Antivachis & Angelis, 2015, p. 589; Provan & Kenis, 2008, p. 244; Raab *et al*., 2015, p. 482).

Provan & Kenis (2008, p. 234) discuss two opposing dimensions of governance: brokered networks and shared governance. Brokered networks are highly centralised and managed by a network broker. In such cases, the network will be governed by either a lead organisation within the network or a network administrative organisation that exists as a separate entity. The authors state that shared governance may not be an appropriate form of governance if the interdependent task requirements are high, as the demands may be too much for network participant members to lead ( Provan & Kenis, 2008, p. 238). In the lead organisation or network administrative governance model, it may be more appropriate to serve and develop the specific competencies to manage the tasks at the network level ( Antivachis & Angelis, 2015, p. 589; Provan & Kenis, 2008, p. 238).

Governance may be linked to effectiveness. Mandell & Keast (2008, p. 721), claim that effectiveness at the organisational level can be expressed as the customer's prosperity, knowledge transfer, and better results for the organisations that are part of the network. The effectiveness of management at the organisational level can therefore be framed by measuring the outputs of the network. Based on Provan & Kenis (2008, p. 237), the success of adopting a particular form of governance relies on four key structural and relational aspects: trust, size of the network, common goals, and the nature of the task ( Antivachis & Angelis, 2015, p. 589). The success of a network depends on leadership taking on different forms in moving a network forward through governance structures and processes ( Bryson *et al*., 2006, p. 52). To unite and integrate a diverse group of members within a network administrative organisation, network leaders must first realise that organisations differ in various factors including size, cultures, values, and more ( Muradli & Ahmadov, 2019, p. 1265).

***Category 2.3: management.*** Muradli & Ahmadov (2019, p. 1257) state that managing inter-organisational networks is a very difficult task. The nature of the tasks stems from two premises: the organisational nature of the activities and the management of resources ( Järvensivu & Möller, 2009, p. 9). The management roles perspective mentions that, at the micro-level of analysis, managers seem to be engaged in various roles: interpersonal, informational, and decisional ( Järvensivu & Möller, 2009, p. 9).

Both inter- and intra-network functioning are aimed at value management ( Järvensivu & Möller (2009, p. 3). As a result, the four classic functions of intra-organisational management, namely planning, organisation, leading, and controlling, may be relevant to inter-organisational functioning ( Järvensivu & Möller (2009, p. 3).

Järvensivu & Möller (2009, p. 25) also suggest that there are three managerial roles: architect, lead operator, and caretaker. Whatever the managerial role, Järvensivu & Möller (2009) state that there are four key requirements of management. The first is to ensure a system that creates value and how to attain it. The second requirement is to structure resources, activities, and actors to be able to bring about the value. With the third requirement, management ensures that the actors are mobilised and energised to carry out the activities necessary to add value and finally the fourth requirement calls for the management to check that the value created by the system is what was needed and take measures to improve if not.

***Category 2.4: funding.*** Funding is a key challenge in any organisation, as it allows for the running of day-to-day functions of the network, including communication and organising services for members ( Göbel *et al*., 2016, p. 39). Resources, such as funding, that are available at the network or community level also contribute towards network effectiveness ( Provan & Milward, 1995, p. 27). For example, conflict within a network can be exacerbated due to a lack of funding ( Bryson *et al*., 2006, p. 48).

Three typical sources of funding, seen in citizen science networks, include membership fees, grants, and donations ( Göbel *et al*., 2016, p. 39). While membership fees provide a stable form of income to make networks sustainable, they are not the best source of funding in the initial phases of setting up the network ( Göbel *et al*., 2016, p. 39). Not all networks have a membership structure from the very beginning, so membership fees may be considered at a later stage of development. Associations vary in their approaches, though: the European Citizen Science Association (ECSA) began with a paid membership structure, while the Citizen Science Association (CSA) and the Australian Citizen Science Association (ACSA) incorporated membership fees later on ( Göbel *et al*., 2016, p. 16).

Research grants are another important source of funding for example in citizen science and public engagement networks ( Göbel *et al*., 2016, p. 39). However, these funding sources vary with changes in public policy. At the end of the European Commission funding programme ‘Science with and for Society’ (SwafS) in 2020, citizen science and public engagement networks had to seek other sources of funding. According to the European Commission participant portal, the funding schemes to engage stakeholders are now an integral part of citizen science projects within the new Horizon Europe Framework programme. Managing funding opportunities is a crucial task and adds more challenges to the governance of networks, especially newly formed organisations.

Other important sources of funding are sponsorship and donations ( Göbel *et al*., 2016, p. 39). This form of funding is more stable than research project funding. However, to be sustainable, a network needs to build a diverse funding portfolio ( Göbel *et al*., 2016, p. 39).

Even though funding is crucial, however, this alone does not ensure favourable outcomes, especially when factors at the network level are also important for the success of the network ( Provan & Milward, 1995, p. 27). In health and human services such as chronic conditions in mental health, the success of the network outcomes rely on other factors such as the density of community development for the network to be successful ( Provan & Milward, 1995, p. 27). Together with governance and management, funding provides a structural link to network effectiveness.

### Theme 3: network relationships

Network relationships include the categories of membership (focused on diversity and inclusion), the category of actors and stakeholders, (which reflects literature on trust), and relationships as a category, (focused on negotiations of influence of power).

***Category 3.1: membership.*** Gender and diversity are key aspects within RRI policy ( EC, 2020b, Dec 7), and SDGs ( UNESCO, 2015) and so are one of the prevalent dimensions to consider when measuring network effectiveness. Diversity of participation is a necessary dimension; Isett *et al.* (2011, p. 166) argue that it must not be neglected when creating formal networks. While network theory does not explicitly tackle gender, diversity, and inclusivity, Acker (2006, p. 442) suggests that organisations that have practices that are not entirely inclusive, or which neglect gender issues, result in further disparity and inequalities between gender, racial, and class within their networks.

Innovation is crucial to research, including science and technology. Innovation may be defined as the generation and successful implementation of new ideas ( Axtell *et al*., 2000, p. 269). Being innovative requires being able to create and implement ideas and go beyond their initial state ( Foss *et al*., 2013, p.300). To support innovation processes, Axtell *et al.* (2000, p. 281) argue that networks need to include a diversity of actors. Levén *et al.* (2014, p.160) argue that including diverse actors adds value to network members and is important to being successful and effective.

Levén *et al.* (2014, p. 165) suggest that the challenge is to ensure a diversity of stakeholders at the level of network membership. Bryson *et al.* (2006, p. 48) propose making use of stakeholder analyses to increase the possibility of success in cross-sector collaborations. In turn, this builds trust and the capacity to manage conflict while building on the competencies of collaborators.

In the articles included in this review, the role of gender and diversity was not explicitly addressed. Gender-related management capacities, such as initiatives taken to address diversity and gender issues, are currently missing.

***Category 3.2: stakeholders and actors.*** Trust and types of trust between stakeholders and actors emerged as central to the effectiveness of networks ( DeWever *et al*., 2005, p. 1526). Trust may rely on confidence in the network’s administration and performance, together with interpersonal behaviours ( Bryson *et al*., 2006, p. 48). Trust can also rely on confidence in organisational competence as well as a sense of goodwill ( Chen & Graddy, 2010, p. 408).

The core of collaborative work lies in trusting relationships ( Bryson *et al*., 2006, p. 47). Varying levels of trust are needed in collaborations and this is an ongoing process ( Antivachis & Angelis, 2015, p.589; Bryson *et al*., 2006). Lack of trust can induce suspicion and impede collaboration ( Auschra, 2018, p. 7). With no pre-existing relationships, collaborations between partners usually build slowly and gradually over small projects where the level of trust needed is not high ( Bryson *et al*., 2006, p.46). DeWever *et al.* (2005, p. 1526) also state that network effectiveness is dependent on the relational dimension of social capital, conceptualised as trust. Therefore, trust can leave an indirect or direct effect in acquiring resources in inter-organisational networks ( DeWever *et al*., 2005, p. 1529). DeWever *et al.* (2005, p. 1526) identify three aspects of trust that affect network effectiveness: different types and levels, as well as the interaction between trust and other variables that are crucial to analyse network effectiveness. There needs to be a balance of trust, otherwise in the case of a company wanting to purchase a product, an imbalance of trust results in reduced motivation for negotiators to purchase from that supplier ( Jeffries & Reed, 2000, p.879). Elmi *et al*. (2013) view trust in inter-organisational networks as an internal resource. Trust should be kept at an optimal level ( Jeffries & Reed, 2000, p. 880). Managers and orchestrators within a network have the core task of providing a foundation for network members to develop trusting relationships ( DeWever *et al*., 2005, p. 1525; Reypens *et al*., 2019, p. 11). Trust is also a key characteristic in network management. The network administrative team is to resist imposing too much central control but still encourage members’ engagement and dissemination of both information and resources ( Reypens *et al*., 2019, p.5). DeWever *et al.* (2005, p.1536) argue that a high level of trust is to be avoided as it can lead to a very rigid system or lost opportunities for less trusting partners/stakeholders in acquiring strategic resources.

Human & Provan (1997, p. 398) state that one of the necessary and distinct dimensions critical for networks are interactions that can build trust among network members. This is necessary to be able to communicate within the network.

Two dimensions of trust are resiliency and specificity, which DeWever *et al.* (2005, p. 1530) develop into a matrix of different types of trust, along dimensions of fragility and resilience. The level of resiliency is classified as either fragile or resilient: the more resilient the trust is between members and in the network, the less information is needed to accept collaboration. Specificity relates to the level of trust that exists in a network without the need for previous information or interaction. The degree of specificity is further explained on two levels: dyadic trust or generalised trust ( DeWever *et al*., 2005, p. 1529). Factors that influence trust include affiliation and/or reputation of members or other networks that contribute to the concept of trustworthiness ( Barney & Hansen, 1994, p.187).

The screened literature illustrates the crucial role that trust has in the relational dimension of stakeholders and actors’ network effectiveness, both at the community and network levels. Two important dimensions of trust are explored in DeWever *et al*. (2005, p. 1529)’s analysis: resiliency and specificity. The type of trust depends on the level of resiliency of trust; fragile or resilient ( DeWever *et al*., 2005, p. 1530).

***Category 3.3: relationships.*** The included literature reflected a discussion on the relationships between individuals within networks, especially when it comes to power dynamics. In networks, power has been explained as the capacity of actors to intentionally influence the actions and behaviours of others ( Provan & Sydow, 2009, p. 16). Causal powers are based on the structure of the network ( Järvensivu & Möller, 2009, p. 10 citing Tsoukas, 1994). Benson (1975, p. 247) views the dimension of the organisation's resources based on the analysis of the superstructure and the substructure of the network. The superstructure is defined by the interactions between actors in a network while the substructure focuses on the role of power in a network ( Benson, 1975, p. 247). Imbalances of power in relationships in networks are conducive to mistrust and affect collaboration ( Bryson *et al*., 2006; Huxham & Vangen, 2005, p.173). A cause for an imbalance of power in networks is disagreeing on a shared purpose ( Bryson *et al*., 2006, p. 50). Bryson *et al.* (2006, p.50) argue that there is a greater chance of success at cross-sector collaborations when resources and tactics are used in dealing with power imbalances. Conflicts and power imbalances may act as barriers to inter-organisational collaboration and affect collaboration outcomes ( Auschra, 2018, p. 7). Power imbalances may also result in defence of resources and authority by individuals, leading to power conflicts within networks ( Auschra, 2018, p. 7).

The categories within relationships focus our attention on inclusion and diversity, trust, and negotiations of power within relationships. These categories are linked to innovation, collaboration, and, in negative cases, defensiveness.

## Discussion and conclusion

In this scoping review, we explored the theoretical and substantive insights into networks which were relevant to implementing an RRI approach in achieving the SDG principles, focused on implications for building and sustaining an effective and sustainable inter-organisational network. Studies reviewed under the first theme captured overlap of terms between effectiveness and sustainability and success and sustainability. The factors that contribute to effectiveness, sustainability and the success of networks include the age of the network, funding, the type/function of network, the services offered, and the structural and relational aspects of monitoring and evaluating the network. However, the terms ‘function’ and ‘types’ are used interchangeably in network studies ( Popp *et al*., 2014, p.30). The function may vary depending on the outcome that particular networks want to achieve, and networks may have more than one function ( Göbel *et al*., 2016, p. 12; Provan & Kenis, 2008, p. 230).

Muradli & Ahmadov (2019, p. 1257) state that managing inter-organisational networks is a very difficult task. Järvensivu & Möller (2009, p. 18/19) assert four key requirements of management that may be applied to networks to create and attain value and improve. We suggest that management’s role at the organisational level can be used to measure the effectiveness of the network through measuring its outputs.

The screened-in literature highlights three types of governance models. The lead organisation or network administrative governance model may be more appropriate to serve and develop the specific competencies of managing the tasks at the network level ( Antivachis & Angelis, 2015, p. 589; Provan & Kenis, 2008, p. 238). Network level analysis explores the entire network and not simply the organisational level. Creating a successful governance structure depends on trust, size of the network, common goals and the nature of tasks ( Provan & Kenis, 2008, p. 237). Furthermore, success also relies on appropriate leadership in choosing the right governance system, managing relationships with members and choosing appropriate stakeholders ( Bryson *et al*., 2006, p. 52).

Both governance and management mechanisms need to be chosen with consideration to have an effective network, be it participant-governed, lead-organisation, or through an administrative organisation external to the network, especially in the initial phases. The scoping review indicates that the choice of governance is dependent on the type/function of the network. In addition, understanding the goals that network members want to achieve informs how governance can proceed in sustaining the network. Having a diverse funding portfolio, schemes, and other resources are also asserted as necessary for network effectiveness ( Göbel *et al*., 2016, p. 39). Networks must address issues involving complex coordination, funding, management, conflict, and power issues.

The included studies highlighted issues within the third theme, ‘relationships’, of power in any form, at network or community level, as well as demonstrating a lack of literature within network theory that addresses gender, diversity and inclusivity. Imbalances in power may cause mistrust between partners and disrupts collaborations, suggesting the importance of managing relationships and power dynamics at the network level between stakeholders and between members of the network. This implies that although the management style that is selected depends on the choice of governance, the leadership style needs to be flexible to adapt according to members’ needs. Incorporating factors in the overall membership to ensure diversity of members and stakeholders in terms of gender and knowledge is crucial. These findings suggest that when setting up such a network, culture should be taken into consideration. This may be very relevant to the interpretations of RRI national priorities when implementing SDG principles. Maintaining inclusion and diversity throughout the existence of a network may also help to ensure its effectiveness and sustainability in the long term.

Although not a direct focus of the review, the included articles demonstrate that networks are often difficult to form and sustain. Securing funding such as donations, membership fees, grants, and other modes helps to ensure a networks’ sustainability so that the network can be managed and provide the necessary services for its members. In the early stages of network formation, internal and external legitimacy and support are important considerations. The external environment and resources are vital determinants to ensure network sustainability. Ongoing evaluation and monitoring needs to be put in place to oversee whether the network is functioning at both the network level and for its members, based on the aims and services it promised to its stakeholders and members. This may help to ensure the network’s sustainability and success.

Limitations of this review reflect restricted/paid access to certain journals and/or articles to research articles, using only one academic database, and only including articles written in the English language. Articles on network theory and networks were chosen from a wide spectrum of fields, with great variation in context and backgrounds, with diverse methodologies. Having such a mix of methodologies could prove fruitful in applying network theory to a specific network in practice, moving beyond theory and applying it in a practical context. 

### A way forward

Our review confirmed that no specific literature exists on the development of inter-organisational networks for implementing an RRI framework or SDG principles. However, lessons learned from networks in other sectors and in network theory may further inform ways to be effective in the long-term, by incorporating diverse inter-sectoral actors working together to implement RRI/SDG goals. Furthermore, given the complexity of integrating RRI and SDGs in a global context, a lead-organisation or administrative organisation external to the network might prove better to coordinate a large network. However, further research is needed to better understand how negative experiences at network and community levels impact networks, and why certain networks thrive while others perish. Further scoping reviews in other languages, besides English, and other national and cultural contexts could add value in addressing the global complexity of networks and their potential operation as RRI and SDG networks. These networks could determine a benchmark for a common vision, mission and objectives and build a shared global purpose that can monitor and evaluate the impact of research and technology. Stakeholder analysis is recommended to ensure that a diversity of stakeholders, inclusive of multiple diversities such as race, gender, ethnic backgrounds or other diversities are present and included within a network. Further research could also explore the impact of diversity on being innovative and effective, specifically in connection with network governance and management at network and community levels. ANT may help identify both non-human and human actants that should be considered with an RRI framework in achieving the SDGs. While network theory and analysis framed our scoping review, an area of further analysis could look into how communities of interest or practice operate in the context of RRI and the SDGs.

One of the goals of a scoping review may be to examine the terminology used in a field ( Grant & Booth, 2009, p. 93). A prevalent issue and finding in the included literature was the multiple meanings assigned to various terms, such as the interchange between types and functions of networks, governance and management, as well as the overlap between approaches to understanding the effectiveness, sustainability, and success of a network. Future research should delve into the different facets and interplay of these terms and their use within network studies.

We suggest further empirical research is needed to link effectiveness, sustainability, and success of how networks can engage stakeholders at various levels of the research process. Evidence on the role of gender and its peer effects is scarce in network analysis, and should be researched to also explore the intersection with racial and class inequalities within organisations. Conducting empirical research on network evolution and evaluation may inform how inter-organisational networks for RRI approaches to the SDGs may be effective, sustainable and successful. We suggest generating an evaluation framework that provides a set of indicators that can indicate whether a network is successful. These indicators will inform how building such a community can be sustained in the long term and how best to evolve it. This will also help to understand how the function of inter-organisational networks may vary depending on the specific role of the network.

The findings suggest that working toward a global coalition to implement RRI/SDGs should emerge in interaction with members’ requirements and be flexible towards evolving needs and expectations. As there are various definitions and potential regional differences on RRI, and various approaches taken when addressing national contexts in applying SDG principles, a starting point is to discuss a common vision, mission, and objectives. This assists in establishing the value of a network to its members. Many of the aims associated with the formation of a global network to address the SDGs include allowing organisations to be responsive to change while allowing the flexibility of innovation. The findings indicate that a shared purpose, such as clarity of aims, reduces the imbalance of power in the network.

Networks have been shown to strengthen the collaboration among various stakeholders and sectors, which shows promise in aligning the pillars of RRI with the SDGs. Other benefits of networks include enhancing knowledge sharing among practitioners, researchers, citizens, and other stakeholders. Furthermore, as highlighted in the literature, networks have the potential to empower communities to respond to societal challenges with greater resilience.

## Data availability

### Underlying data

All data underlying the results are available as part of the article and no additional source data are required.

### Extended data

Zenodo: PRISMA_Flowchart Farrugia et al 2021_Effective inter organisational networks for RRI and global sustainability. https://doi.org/10.5281/zenodo.5550321 ( Farrugia *et al*., 2021a)

This project contains the following extended data:

PRISMA_Checklist Farrugia et al 2021_final.pdf (PRISMA-ScR Flow Chart for RRI Network Studies)

### Reporting guidelines

Zenodo: PRISMA-ScR checklist for ‘Effective inter-organisational networks for Responsible Research and Innovation and global sustainability: A scoping review’. https://doi.org/10.5281/zenodo.5592147 ( Farrugia *et al*., 2021b)

Data are available under the terms of the Creative Commons Attribution 4.0 International license (CC-BY 4.0).
