# Supplementary material for: Effective inter-organisational networks for Responsible Research and Innovation and global sustainability: a scoping review
Source: Open Res Eur. 2022 Jul 20;1:144. Originally published 2021 Nov 30. [Version 2] doi: 10.12688/openreseurope.13796.2 (PMC10446013; doi:10.12688/openreseurope.13796.2)
Supplement: Peer review history

# Peer review: Reviewer response for version 2

**DOI:** 10.21956/openreseurope.16048.r29733

**Published:** 2022-08-15

**Authors:** Alice MacGillivray

**Affiliations:** 1 Royal Roads University, Victoria, Canada

Comments well addressed.

Are sufficient details of the methods and analysis provided to allow replication by others?

Partly

Are the rationale for, and objectives of, the Systematic Review clearly stated?

Partly

Is the statistical analysis and its interpretation appropriate?

Yes

Are the conclusions drawn adequately supported by the results presented in the review?

Yes

Reviewer Expertise:

Complexity, leadership, sustainability.

I confirm that I have read this submission and believe that I have an appropriate level of expertise to confirm that it is of an acceptable scientific standard.

# Peer review: Reviewer response for version 2

**DOI:** 10.21956/openreseurope.16048.r29732

**Published:** 2022-07-21

**Authors:** Penny Haworth

**Affiliations:** 1 South African Institute for Aquatic Biodiversity Penny Haworth (SAIAB), Grahamstown, South Africa

I am happy that my comments have been addressed. Thank you for the opportunity to respond

Are sufficient details of the methods and analysis provided to allow replication by others?

Yes

Are the rationale for, and objectives of, the Systematic Review clearly stated?

Yes

Is the statistical analysis and its interpretation appropriate?

Yes

Are the conclusions drawn adequately supported by the results presented in the review?

Yes

Reviewer Expertise:

Museum Education, Science Communication and RRI

I confirm that I have read this submission and believe that I have an appropriate level of expertise to confirm that it is of an acceptable scientific standard.

# Peer review: Reviewer response for version 1

**DOI:** 10.21956/openreseurope.14875.r28922

**Published:** 2022-04-05

**Authors:** Penny Haworth

**Affiliations:** 1 South African Institute for Aquatic Biodiversity Penny Haworth (SAIAB), Grahamstown, South Africa

To my mind, this scoping review does what it sets out to do. The limitations of the review are acknowledged and openings for further research are suggested.

1. Given the limitations (restricted/paid access to journals and articles, the use of only one academic database and the review of only articles written in English – p.13), an additional recommendation may be that further scoping reviews, especially in contexts other than Europe, and languages other than English,  could be of value.  Perhaps such future scoping reviews could also be useful in determining a benchmark for, or to establish, a “common vision, mission, and objectives” (p.14) and may provide a starting point from which to build a shared global purpose and through which to monitor and evaluate impact.
2.  Whilst implicit in the article, perhaps it could be more clearly articulated that “global” concerns include the promotion of RRI in the global South where similar efforts are being made to embed RRI in the science agenda. As mentioned in the article, definitions of RRI may vary (p.15).
3. Note: With regard to the extended date range used for inclusion of works of early scholars (1990-2020 - p.5), there seems to be an anomaly, as the literature cited includes a reference to Benson:1975 (p.12) and References p.14.

Are sufficient details of the methods and analysis provided to allow replication by others?

Yes

Are the rationale for, and objectives of, the Systematic Review clearly stated?

Yes

Is the statistical analysis and its interpretation appropriate?

Yes

Are the conclusions drawn adequately supported by the results presented in the review?

Yes

Reviewer Expertise:

Museum Education, Science Communication and RRI

I confirm that I have read this submission and believe that I have an appropriate level of expertise to confirm that it is of an acceptable scientific standard, however I have significant reservations, as outlined above.

# Peer review: Reviewer response for version 1

**DOI:** 10.21956/openreseurope.14875.r28117

**Published:** 2022-02-08

**Authors:** Alice MacGillivray

**Affiliations:** 1 Royal Roads University, Victoria, Canada

First, I apologize for taking so long and for writing a brief report. I am commenting as I read, which I realize has drawbacks as well as advantages.

 I think the topic is timely, important, and exciting.

 I chose "partly" for the first item because it asked about a systematic review, when this is a scoping review.

 I did find the abstract a bit confusing. I expected a question--phrased as such--to guide the review. The scope felt huge. SDGs cover so many topics as do topics such as networks and communities of interest and practice (CoP). When science, social science, culture and politics are added and wondered about inclusion and exclusion criteria. I know these were articulated later. I like the idea of aligning with initiatives in motion. In the early part of the paper, it almost felt as if the whole paper could be an interesting critique of SDG/RRI framing.

 It becomes apparent that network theory is framing ideas in the paper. This is a bit different than the social learning theory behind communities of practice. Although I'd love to see a paper with another methodology in which CoP working with SDGs are sought out, I'm wondering if the CoP concept should be left out to keep the focus on networks and network theory. Almost by definition, CoP work does not often make it into academic literature.

 The "analytic approach" paragraph helps to clarify many of my initial questions. However, I was still not quite clear on whether the paper was exploring unrealized possibilities or elements of existing practical approaches to networking for SDG work. Again, this is a comment related more to clarity of presentation than value of work. It becomes clear later, but I would have loved clarity right from the abstract through the paper.

 As a sidebar comment, I agree that managing inter-org networks is very difficult. I also acknowledge they can thrive if they operate driven by passion (as in a CoP) under the radar. But again, that might not be captured in a scoping review.

 I am reminded of different governance models including evolutionary governance theory as I read "a way forward." 

 I'm not sure how helpful this has been. As mentioned, I think this is a very important topic and I wish more of the passion I feel about the topic had come through in its presentation. There are urgent needs, insights from network theories (and communities of practice and governance theories and practice--though out of scope). But style choices should be determined by editors of course. I think it should pass peer review with some edits.

Are sufficient details of the methods and analysis provided to allow replication by others?

Partly

Are the rationale for, and objectives of, the Systematic Review clearly stated?

Partly

Is the statistical analysis and its interpretation appropriate?

Yes

Are the conclusions drawn adequately supported by the results presented in the review?

Yes

Reviewer Expertise:

Complexity, leadership, sustainability.

I confirm that I have read this submission and believe that I have an appropriate level of expertise to confirm that it is of an acceptable scientific standard, however I have significant reservations, as outlined above.
